# Transcriptional and phenotypical heterogeneity of *Trypanosoma cruzi* cell populations

**DOI:** 10.1098/rsob.150190

**Published:** 2015-12-16

**Authors:** Víctor Seco-Hidalgo, Luis Miguel De Pablos, Antonio Osuna

**Affiliations:** 1 Biochemistry and Molecular Parasitology Research Group, Department of Parasitology, University of Granada, Campus de Fuentenueva, Granada, Spain; 2 Centre for Immunology and Infection (CII), Biology Department, University of York, York, UK

**Keywords:** kinetoplastid, gene expression, RNA, protozoan, mucin-associated surface protein

## Abstract

*Trypanosoma cruzi* has a complex life cycle comprising pools of cell populations which circulate among humans, vectors, sylvatic reservoirs and domestic animals. Recent experimental evidence has demonstrated the importance of clonal variations for parasite population dynamics, survival and evolution. By limiting dilution assays, we have isolated seven isogenic clonal cell lines derived from the Pan4 strain of *T. cruzi*. Applying different molecular techniques, we have been able to provide a comprehensive characterization of the expression heterogeneity in the mucin-associated surface protein (MASP) gene family, where all the clonal isogenic populations were transcriptionally different. Hierarchical cluster analysis and sequence comparison among different MASP cDNA libraries showed that, despite the great variability in MASP expression, some members of the transcriptome (including MASP pseudogenes) are conserved, not only in the life-cycle stages but also among different strains of *T. cruzi.* Finally, other important aspects for the parasite, such as growth, spontaneous metacyclogenesis or excretion of different catabolites, were also compared among the clones, demonstrating that *T. cruzi* populations of cells are also phenotypically heterogeneous. Although the evolutionary strategy that sustains the MASP expression polymorphism remains unknown, we suggest that MASP clonal variability and phenotypic heterogeneities found in this study might provide an advantage, allowing a rapid response to environmental pressure or changes during the life cycle of *T. cruzi*.

## 1. Introduction

*Trypanosoma cruzi* is a flagellate protozoan parasite belonging to the order Kinetoplastidae and is the aetiological agent of Chagas' disease, a major public health problem in Central and South America. Although this parasitaemia has been traditionally confined to Latin America, cases have been diagnosed outside this area because of human migration from endemic zones [1–7]. Thus, currently there are more than 8 million people infected with approximately 25 million people at risk of acquiring the disease, making it a significant problem for global public health worldwide with an estimated annual burden of $627.46 million in healthcare costs and 806 170 DALYs (disability-adjusted life years) [8–10]. This flagellate needs a mammalian host and an insect vector to complete its life cycle. In the intestinal tract of the insect (family Reduviidae, subfamily Triatominae), the bloodstream trypomastigote forms, ingested from the mammalian host, transform into the replicative and non-infective epimastigote forms. After approximately 8–15 days, the epimastigote forms develop into metacyclic trypomastigotes in the rectum of the triatomine. These metacyclic forms, which are not replicative, are transmitted during the insect blood meal with the faeces and urine infecting mammalian host cells through the bite wound or the surrounding mucosal membranes. Once host cells are infected, the parasites transform into amastigotes, which is the intracellular replicative form. The amastigote forms multiply and differentiate into bloodstream trypomastigote forms, which burst out of the cell and are liberated into the intercellular spaces and the bloodstream, being disseminated throughout the host. The circulating parasites can then invade new cells and initiate new replicative cycles, and they are available to infect vectors that feed on the host.

The genetic variation of the natural populations of *T. cruzi* has been widely studied [11–15]. Digging deeper into this diversity, recent advances in individual cell analysis revealed the importance of considering cellular populations as a complex mosaic of cells where cell to cell heterogeneous processes happen under the same deterministic genetic programme [16].

There are several environmental conditions that can change during the course of the *T. cruzi* life cycle such as immune and drug pressures, host genetics, the presence and magnitude of febrile episodes, host metabolic and nutritional conditions [17–19], and even the presence of competing parasites [20,21] or bacterial microbes [19,22]. The rapid adaptation to environmental changes is essential for parasite survival and to this end phenotypic mosaicism might offer a selective advantage in responding quickly to these changes and establishing a robust chronic infection, as has been described in *Plasmodium falciparum* and *Leishmania* spp. [16,23,24].

A remarkable feature of *T. cruzi*, in comparison to other kinetoplastids such as *Leishmania major* or *Trypanosoma brucei,* is the dramatic expansion of several families of surface molecules located in non-syntenic ‘islands’ of the *T. cruzi* genome (i.e. where gene order is non-conserved among the three trypanosomatids). Importantly, these ‘islands’ were found to contain the multigene family of mucin-associated surface proteins (MASPs) which, with 1400 members, are present in high numbers on the surface of the parasite and correspond to approximately 6% of the parasite diploid genome [25,26]. The MASP family is characterized by having highly conserved N- and C-terminal domains and a variable and repetitive central region, with a maximum expression in the human infective stages of the parasite [27,28]. It is thought that the MASP family plays an important role in the invasion of the mammalian host cell [28–30], but could also be crucial for the survival and the establishment of the parasite in the invertebrate host as demonstrated for the mucin family of proteins of *T. cruzi* [19,31].

Although, some reports have shown the presence of clonal and inter-strain karyotypic differences and copy number variations on multigene families including MASPs [28,32,33], this clonal heterogeneity remains incompletely characterized.

In our research, several molecular-based techniques have been applied on clonally isogenic populations of *T. cruzi* cells to search for and measure variations in cellular processes such as RNA transcription and protein translation of the MASP family. Other phenotypic aspects such as growth dynamics, spontaneous metacyclogenetic ability or variation in the catabolites produced by these isogenic lines were also analysed.

## 2. Material and methods

### 2.1. Cell and parasite cultures

Host Vero cells (ECACC 84113001) were cultivated at 37°C (pH 7.2) in a moist atmosphere with 5% CO_2_ in 75 cm^2^ plastic flasks (Nunc) containing Dulbecco-modified Eagle medium (DMEM; Gibco) supplemented with 10% (v/v) heat-inactivated fetal calf serum (56°C for 30 min) (IFSC, Gibco).

The epimastigote forms of CL Brener (TcVI), Pan4 (TcI) and Maracay (TcI-II) of *T. cruzi* strains were regularly maintained in logarithmic growth phase at 28°C in RPMI-1640 medium (Sigma) supplemented with 10% IFSC. The metacyclic trypomastigote forms were obtained in modified Grace's medium with 10% IFSC and purified according to the method described by Osuna *et al.* [34,35]. Cultures of Vero cells were infected with the metacyclic trypomastigotes as described previously [34], being cell-derived trypomastigote forms collected from cell culture supernatants at 96 h post-infection and purified by centrifugation in a discontinuous Percoll density gradient (1.070, 1.065 and 1.060 g ml^−1^) prepared as described elsewhere [36]. Only the forms of the biological cycle of *T. cruzi* that had greater than 90% purity were used, as checked by Giemsa staining.

### 2.2. *Trypanosoma cruzi* cloning

The cloning of the Pan4 strain of *T. cruzi* was carried out by limiting dilution. For this procedure, we used a Pan4 strain that was originally isolated from a 32-year-old patient from the Arraijan district (Panamá) in 2006, being immediately cryopreserved and kindly donated by Prof. Argentina Ying. Briefly, an original stock of Pan4 strain was cultured at epimastigote stage in a total of three passages and then transformed to metacyclic stage forms that were used for the infection of Vero cells. To maximize the diversity within the culture, we performed three consecutive rounds of infection, being the supernatant with trypomastigote parasites collected, centrifuged at 300*g* for 10 min at 4°C and finally cultured as epimastigotes for two passages. Then, the cells were resuspended at a concentration of 1000 parasites ml^−1^ and 1 µl aliquots containing one organism were placed in a 96-well cell culture Microplate (Nunc™), leaving empty wells between all the sample wells to avoid cross-contaminations. Only wells containing single cells were selected and cultured as described above for further experiments. A total of seven clones were obtained by this method and cultured for two passages before the experiments. All further experiments after the cloning were carried out using the same number of parasite passages (three passages) and keeping the same cell densities to avoid any possible off-target effects.

### 2.3. Growth curves and metacyclogenesis

To determine the growth curve of the parental parasite cell line and all the subclones, three biological replicates from each cell line were seeded at day 0 with a concentration of 1 × 10^6^ epimastigotes ml^−1^ and counted using a Neubauer chamber every 24 h for a total of 11 days. Metacyclogenesis was studied by counting the number of metacyclic trypomastigotes which appear spontaneously at the stationary phase (days 9–11) of the epimastigote growth curves mentioned above [34,35].

### 2.4. RNA isolation, cDNA synthesis and quality control

Total RNA was extracted using the RNeasy minikit (Qiagen, Hilden, Germany) and further treated with DNaseI to avoid any DNA contamination. The mRNA was purified from the total RNA extractions using the Oligotex mRNA kit (Qiagen, Hilden, Germany). The cDNA samples were generated using oligo(dT) primer mix from iScript Select cDNA synthesis kit (Bio-Rad Laboratories, Inc.), and the concentration and purity of the RNAs and cDNAs were measured in a Nanodrop ND-1000 spectrophotometer (Thermo Scientific). The quality of the cDNA was tested by PCR using specific primers for aromatic l-alpha-hydroxy acid dehydrogenase (AHADH2) (AHADH2_F 5′ CCAAATGTTTCGCCACTCG 3′ and AHADH2_R 5′ CACGCTGCGGAGGGATCTC 3′) [29]. Each of these PCR reactions was done using 300 ng of cDNA and 400 ng of mRNA (negative control for DNA contamination) with 200 µM dNTPs, 2 mM MgCl_2_, 1 mM primers and 1 U Taq DNA polymerase (MBL) in 1× MBL PCR buffer.

### 2.5. MASP expression libraries

For MASP transcript amplification, specific primers were designed by submitting 250 *masp* members from the CL-Brener *T. cruzi* genome [37] to a motif search by MEME [38]. The most represented motifs were selected as targets for the primer design and subsequently analysed using BLASTN algorithms [39] to verify the high degree of homology and specificity.

A preliminary inter-strain MASP expression library was obtained by a single PCR from metacyclic trypomastigotes of different *T. cruzi* strains (Pan4, Maracay and CL Brener) using the primers MASP N-term_F 5′ ATGGCGATGATGATGACCGGC 3′ corresponding to the conserved MASP signal peptide region and MASP C-term_R 5′ CCACCACCGCAGTAGCAG 3′ for the conserved C-terminal region. The amplicons were extracted and purified from low-melting agarose gels (USBiological) and cloned in pGEM-T Easy vectors (Promega).

**Figure 1.**
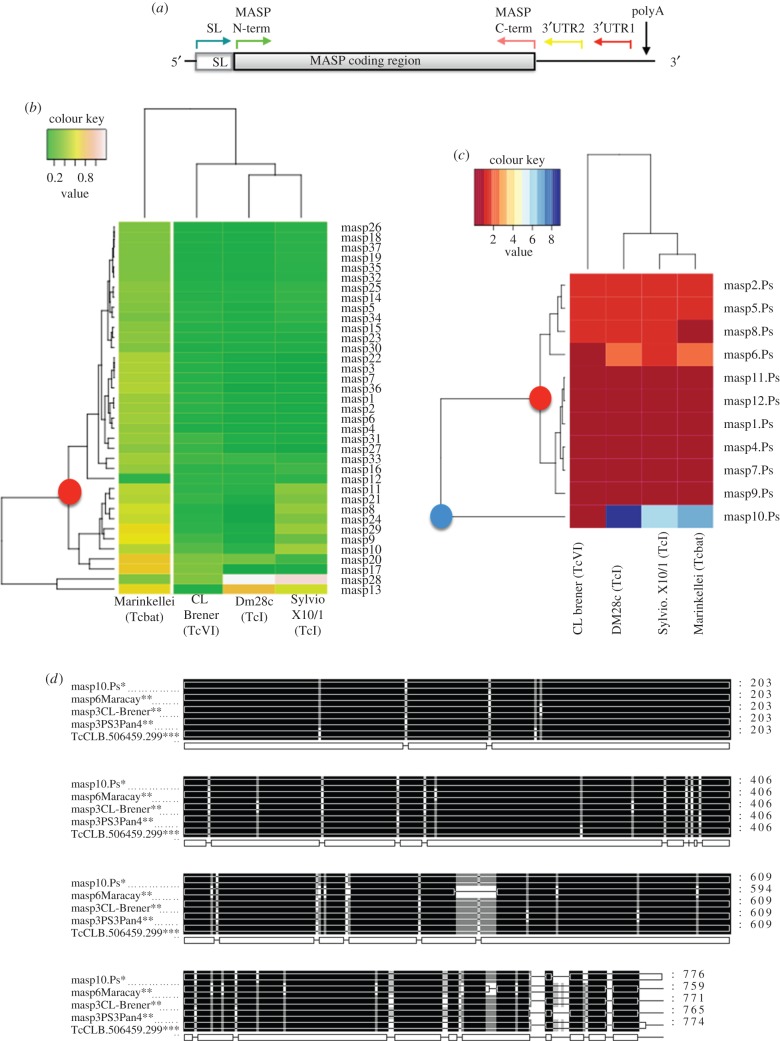
(*a*) Representation of a standard MASP transcript indicating the position of the primers used for the MASP amplification from cDNA by three-step nested PCR. SL, splice leader region; polyA; polyadenylation site. (*b,c*) Hierarchical cluster analyses based on the three-step MASP library according to the pairwise distances with the orthologue sequences of CL Brener, Dm28c, Sylvio X10/1 and Marinkellei strains. Heatmaps were splitted on gene (*b*) and pseudogene (*c*) sequences. The horizontal nodes and their length represent the distance among the MASP members of the three-step MASP library, whereas the vertical nodes and their lengths represent the distances among the outlined strains. In the heatmap of genes, the green colours show the minimum distance, whereas white colours indicate maximum distance. In the heatmap of pseudogenes, the minimum and maximum distances are represented with red and blue colours, respectively. The red dots show the point of divergence between the slow and fast evolving groups of MASP genes and pseudogenes. The blue dot indicates the point of divergence for *masp10.Ps* (*d*) SNP analysis of MASP sequences by Clustal multiple alignment. Complete homology among the sequences is represented with black areas, whereas grey nucleotides represent areas with less than 80% of homology. *Three-step MASP library (trypomastigore stage); **preliminary MASP library (metacyclic trypomastigote stage); ***dos Santos *et al.* MASP library (bloodstream trypomastigote stage) [29].

In order to have a broader spectrum of MASP amplicons, we constructed a three-step MASP library using trypomastigotes of the PAN4. In brief, we followed the semi-nested PCR method described by dos Santos *et al.* [29], but adding a further step of nested PCR with the primers described above for a total of three sequential PCR reactions (three-step MASP library). Thus, the following primer combinations were used: SL 5′ AACGCTATTATTGATACAGTTTCTGTACTATATTG 3′ for the 35 bp spliced leader region and 3′UTR1 (reverse) 5′ GTGTGCTTCGTGGGGTGAGGTG 3′ for the 3′UTR in the first reaction; SL and 3′UTR2 5′ CTCACTCTCACGCGGCCACCACCACCG 3′ also for 3′UTR (internal to 3′UTR1) in the second reaction [29]; and finally MASP N-term_F 5′ ATGGCGATGATGATGACCGGC 3′ (internal to SL) and MASP C-term_R 5′ CCACCACCGCAGTAGCAG 3′ for the conserved carboxyl extreme (internal to 3′UTR2) in the third reaction (figure 1*a*). The amplicons of the third reaction were cloned in pGEM-T Easy (Promega).

Both MASP libraries were constructed in 25 µl PCR reactions using 200 µM dNTPs, 2 mM MgCl_2_, 1 mM primers and 1U Taq DNA polymerase (MBL) in 1× MBL PCR buffer. The inter-strain MASP library was constructed from 500 ng cDNA of each strain. Meanwhile, the nested PCR reactions of the three-step MASP library in Pan4 were elaborated using 600 ng of the cDNA for the first reaction and 3.3 µl of amplified product from the first and second reaction as template DNA for the second and third reaction, respectively, with 200 µM dNTPs, 2 mM MgCl_2_, 1 mM primers and 1U Taq DNA polymerase (MBL) in 1× MBL PCR buffer. After cloning and plasmid purification (PureYield^TM^ Promega), each extraction was amplified by PCR using the inner primers (MASP N-term_F and MASP C-term_R) to discard reactions with more than one amplicon inserted. The selected plasmids with unique inserts were submitted to sequencing at one end in a 3130XL Genetic Analyzer (Applied Biosystems) using T7 (forward) 5′ TAATACGACTCACTATAGGG 3′ and SP6 (reverse) 5′ TATTTAGGTGACACTATA 3′.

### 2.6. Phylogenetic analysis on the three preliminary MASP libraries

MASP sequences used for phylogenetic analysis were obtained from a preliminary inter-strain MASP expression library of the CL Brener, Pan4 and Maracay strains of *T. cruzi*. Multiple global alignment was conducted using ClustalW2 algorithm [40,41] and manually edited in Jalview [42]. The evolutionary distances were computed in Mega 6.06 software [43] using the Maximum Composite Likelihood phylogeny test [44] and the Tamura–Nei model. The bootstrap consensus tree was inferred from 1000 replicates.

### 2.7. Hierarchical cluster analyses on the three-step MASP library

To identify the orthologue sequences of the three-step MASP library of Pan4 in the CL Brener, Dm28c, Sylvio X10/1 and Marinkellei strain B7 genomes (http://tritrypdb.org/tritrypdb/), the algorithms BLASTN (for the pseudogenes) and BLASTP (for the genes) were used [39]. The *e*-value (expected value) cut-off for BLAST searches used was 10^−8^. Subsequently, the orthologue sequences from each strain and the sequences from our Pan4 library were aligned by the ClustalW2 algorithm [40,41] and edited in Jalview [42]. The pairwise distances within each strain were calculated using Mega v. 6.06 [43] with 1000 replications of bootstrap resampling as variance method. The distances were used for the hierarchical cluster analysis to generate boxplots and heatmaps using the gplots package and visualized in the R software platform [45].

In addition, our three-step MASP library was aligned against other cDNA MASP expression libraries: our preliminary inter-strain MASP library and the seven MASP libraries performed by dos Santos *et al.* [29]. The visualization of the genetic variations (SNPs) among the different libraries was realized using GeneDoc software [46].

### 2.8. MASP expression analysis: qRT-PCR, capillary electrophoresis and northern blot

The level of MASP expression was quantified for each clone and the parental strain Pan4 of *T. cruzi* by quantitative RT-PCR (qRT-PCR) amplifying a MASP*-*specific fragment from the conserved 5′ extreme region using the primers MASP N-term_F (sequence outlined above; 5′ ATGGCGATGATGATGACCGGC 3′) and MASP N-term'_R 5′ ACCACAGCACGCACAGGGC 3′. Standard curves were used for the calculation of relative quantity (Rq) values of each sample for each target. The expression was normalized using glyceraldehyde 3-phosphate dehydrogenase (GAPDH) GAPDH_F 5′AGCGCGCGTCTAAGACTTACA 3′ and GAPDH_R 5′ TGGAGCTGCGGTTGTCATT 3′ as housekeeping gene. Reactions were performed in triplicate using SYBR® Green Supermix (Bio-Rad) in a C-1000 thermocycler connected to a real-time CFX96 module (Bio-Rad) under the cycling conditions recommended by the manufacturer (Bio-Rad).

Additionally, each clone and the parental strain were also submitted to the nested PCR reactions described above. The amplicons generated in the third reaction were resolved by capillary electrophoresis in a 3130xl Genetic Analyzer (Applied Biosystems). Peak identification and fragment sizing from the electropherograms were obtained by use of Peak Scanner (Applied Biosystems) and the *in silico* gels were analysed using Fingerprinting II (Bio-Rad) with Cosine Coefficient as the curve-based coefficient.

For northern blot analysis, 3 µg of total RNA was run on a 0.9% formaldehyde–agarose gel, transferred to nylon membranes (MSI 0.45 µm) and subsequently cross-linked. The hybridization step was carried out overnight at 42°C in a pre-hybridization buffer (50% formamide, 5× saline–sodium citrate buffer, 5× Denhart's solution and 0.1% sodium dodecyl sulfate) supplemented with 1.25 µg µl^−1^ deoxyribonucleic acid sodium salt from salmon testes (Sigma). Based on the data obtained in the three-step MASP library, two different probes were used: one with the most variable sequence and the other one with the most conserved. Both probes were obtained by PCR amplification from pGEM-T Easy with the insert primers (MASP N-term_F and MASP C-term_R) plus a gel extraction purification (QIAgen). 18S and β-tubulin probes were used as loading controls. All the probes were labelled with dCTP-α[P^32^] (Perkin–Elmer). Before developing, blots were subjected to sequential stringent washes at 65–68°C. The relative level of RNA was quantified by phosphoimager analysis of the northern blots (normalizing with the aforementioned 18S and β-tubulin probes). Analysis was performed using a Typhoon imager and ImageQuant software (GE Healthcare Life Sciences).

### 2.9. Synthesis of peptides and production of polyclonal antibodies

The peptides synthesized for further protein analyses were based on the MASP predicted protein sequences from the expressed genes in the three-step Pan4 library. To do this, we identified the best motifs using the MEME program [38] ProtScale to search for the hydrophobicity of the peptides (http://web.expasy.org/protscale/), and finally ANTIGENIC (http://emboss.bioinformatics.nl/cgi-bin/emboss/antigenic) for calculating the antigenicity of such peptides. Additionally, prior to the synthesis of the peptides, the BLASTP program was used to ensure the specificity of the sequence of the peptides designed. The peptide sequence synthesized by Genscript was (AESQPAGVSVQDATG) MASP-Ag.

Antiserum against this peptide was raised by intraperitoneal injection of BALB/c mice with 50 µg of MASP-Ag linked to keyhole limpet haemocyanin with Freund's complete adjuvant followed by boosters two and four weeks later with Freund's incomplete adjuvant. The mice were bled two weeks after the final booster with incomplete adjuvant. The serum was called anti-MASP-Ag. Test bleed sera was checked by indirect enzyme-linked immunosorbent assay (ELISA) in multi-well plates coated with 10 µg well^−1^ of the synthetic peptide in 0.1 M bicarbonate coating buffer (pH 8.6). Only polyclonal serums against the synthetic peptides with titres higher than 1 : 1600 were pooled and stored at −80°C until used.

### 2.10. Protein electrophoresis and western blotting

MASP protein expression was analysed by sodium dodecyl sulfate polyacrylamide gel electrophoresis (SDS-PAGE) and western blotting. To extract the total parasite proteins from different stages of the life cycle, 5 × 10^7^ organisms of each stage were centrifuged at 800*g* for 10 min at 4°C and washed three times with 20 mM phosphate buffer, 2.7 mM KCl and 137 mM NaCl (pH 7.4) (phosphate-buffered saline, PBS). Afterwards, the pellets were resuspended in 3 v/w of lysis buffer (10 mM potassium phosphate at pH 7.4, 0.25 mM saccharose, 1 mM EDTA, 0.145 mM KCl, 1% Triton X-100) plus Complete Mini inhibitor protease cocktail (Roche Molecular Biochemicals). After treatment for 10 min at 0°C, the parasites were sonicated also at 0°C for 2 min with 10 s cycles, adjusted to the same protein concentration, and electrophoresed using SDS–12.5% PAGE. To view the total protein profiles, the gels were stained with Coomassie brilliant blue. For western blot analyses, total protein extracts were transferred to polyvinylidene difluoride (PVDF) membranes after electrophoresis (Bio-Rad Trans-blot turbo transfer system) and blocked with PBS, 2% non-fat dry milk and 0.1% Tween-20 (Sigma). MASP proteins were detected on western blots using the anti-MASP-Ag outlined above and diluted at 1 : 200 in PBS and 0.1% Tween-20. The blots were revealed with peroxidase conjugated polyclonal goat anti-mouse immunoglobulins at a dilution of 1 : 1000 (Dako). As a loading control the membranes were incubated with the polyclonal sheep anti-α/β tubulin at a dilution of 1 : 1000 (Cytoskeleton) and revealed with peroxidase conjugated anti-Sheep IgG whole molecule produced in donkey at a dilution of 1 : 2000 (Sigma). The software ImageJ 1.48 was used to quantify the western blot signals [47].

### 2.11. RNA turnover assay

In order to study the turnover of the RNA, RNA synthesis was blocked by the addition of 10 µg ml^−1^ actinomycin D (AD; Sigma) to 10 ml of the culture medium containing a total of 5 × 10^7^ flagellate forms which were harvested at various time points (0, 15, 30 min and 2 h) by centrifugation at 800*g* for 10 min at 4°C. Cells were washed in ice-cold PBS and subjected to RNA extraction process and northern blot analysis as previously described.

### 2.12. Proton nuclear magnetic resonance

Proton nuclear magnetic resonance (H NMR) was used to analyse the metabolic end products of glycolysis produced and excreted during the epimastigote culture. For this purpose, 1 × 10^6^ epimastigotes ml^−1^ from each clone were cultured in 25 ml of fresh medium, and at the fifth day of growth (mid-logarithmic phase) the same number of parasites from each culture were centrifuged at 300*g* for 10 min at 4°C. The supernatants were collected and filtered (0.22 µm pore-size filter) for the subsequent ^1^H NMR. The spectra of protonic [^1^H] NMR were obtained with a 300 MHz (2 channels) VARIAN INOVA UNITY spectrometer operating at 300.13 MHz with dual probe broadband 1H with 5 mm Z gradient. The temperature of the probe was maintained at 27°C. The pulse technique and Fourier transformation was used with 90° pulses and a spectrum of 3287.5 Hz. To eliminate watermarks, the method of presaturation was used, selectively irradiating at the frequency of water for 2.5 s with an interval between pulses of 7.5 s, accumulating 160 free induction decays (FIDs), the total of which was multiplied exponentially with a line broadening of 0.2 before Fourier transformation. The chemical shifts are expressed as parts per million (ppm) downfield from trimethylsilyl propanoic acid.

### 2.13. Statistical analysis

Results were analysed using the Tukey–Kramer test of the GraphPad software package InStat, v. 3.05 (32 bit). Differences among the isogenic parasite lines were considered statistically significant when *p* ≤ 0.05 and extremely significant when *p* ≤ 0.001.

## 3. Results

### 3.1. MASP expression libraries and hierarchical cluster analyses

A preliminary inter-strain MASP expression library was performed using the CL Brener, Pan4 and Maracay strains of *T. cruzi.* In this manner, we amplified, cloned and analysed the sequences obtained of 15 MASP (pseudo)genes belonging to the three mentioned strains (electronic supplementary material, figure S1 and electronic supplementary material, table S1). The phylogenetic analysis of these sequences showed a close distance and relationships among 10 MASPs members, whereas others evolved independently (electronic supplementary material, figure S2). However, the members that have close distances among strains also showed conversions from pseudogene to gene or vice versa, so despite being conserved in sequence they are not necessarily expressed at the protein level, or they are expressed as truncated protein versions (electronic supplementary material, figure S2).

For a deeper MASP expression profile, we performed a three-step nested PCR in the Pan4 strain. The amplification profile in each step of the library construction presented a smear (electronic supplementary material, figure S3*c*), indicating the co-expression of several MASP transcripts with different lengths. To analyse the sequence of those transcripts, the products of the third PCR reaction from the trypomastigote stage were cloned into pGEM-T vector and a total of 62 clones were sequenced. Based on the percentage of valid sequences (complete sequences without indeterminations), 93.5% of the library was considered of a good quality (electronic supplementary material, table S2). The *T. cruzi* (pseudo)genes corresponding to each valid sequence are shown in the electronic supplementary material, tables S3 and S4. The use of a third PCR reaction to perform the library increased the total number of clones and the percentage of MASP identification compared with the original protocol from dos Santos *et. al.* (figure 3*c*). We obtained a total of 50 unique sequences with 49 MASP sequences, 37 genes (75.5%) and 12 pseudogenes (24.5%), plus a gene sequence codifying for a hypothetical protein (not included in the hierarchical cluster analysis). It is interesting to note that within the three-step MASP library, we have found several expressed members of the same size but with different numbers and positioning of single nucleotide polymorphisms (SNPs) in their sequences (electronic supplementary material, figure S4).

The hierarchical cluster analysis shown as heatmaps allowed us to represent the distances of the three-step MASP library, with the closest MASP orthologues identified in CL Brener, Dm28c, Sylvio X10/1 and Marinkellei genomes at the gene and pseudogene level (figure 1*b,c*).

The analysis at gene level showed a correlation between the MASP genes and the phylogenetic origin of the different strains (vertical nodes). As was expected, the strain Marinkellei (outgroup), which is a bat-associated subspecies of *T. cruzi* (Tcbat) [48,49], showed the most distanced MASP pattern, whereas Silvio X10/1 and Dmc28 strains (TcI) were the closest to the Pan4 strain (TcI) (figure 1*b*). The clustering of the individual MASP genes (horizontal nodes) also showed two main groups of MASP sequences that, according to their distances, can be grouped as slow (0.22 ± 0.13) and fast evolving (0.40 ± 0.27) (figure 1*c*). The mean distances of all MASP genes in each strain, represented as boxplots, showed similar values for CL-Brener, Silvio X10/1 and Dmc28 strains, with Marinkellei again being the strain with most distance from the Pan4 library (electronic supplementary material figure S5, table S5).

At the pseudogene level, the cluster analysis showed a loss of correlation with the phylogenetic origin of the sequences (vertical nodes). A singular member in the three-step MASP library was the pseudogene *masp3.ps*, which presented a sequence so different in respect to the rest of the strains that it was impossible to even estimate the distance values and was therefore excluded from the hierarchical study. Thus, 11 MASP pseudogenes were analysed, which according to the distances could be clustered into two different groups as slow (0.24 ± 0.19) and fast evolving (1.15 ± 0.37), with the exception of the member *masp10.ps* (5.52 ± 3.82) (horizontal nodes) (figure 1*c*)*.* This pseudogene showed high distance values with respect to the closest orthologues in Silvio X10/1, Dmc28 and Marinkellei strains, but at the same time, *masp10.ps* presented an enormous degree of conservation in the CL Brener strain, being detected in the MASP libraries performed by dos Santos *et al.* [29] from the cDNA of bloodstream trypomastigotes after two and 10 passages in rhesus monkey epithelial cells (LLC-MK2) and from trypomastigotes isolated from rat myoblast cells (L6) after 14 passages. Additionally, *masp10.ps* also showed high percentages of identity with members of our preliminary libraries in all the strains, with eventual recovering of the complete open reading frame in the case of the Maracay and CL-Brener strains (figure 1*c*,*d*; electronic supplementary material, table S5). Although, in general, the mean pairwise distances were smaller among genes compared with pseudogene MASP sequences, it is interesting to note the distance similarity of the pseudogenes among the five strains of *T. cruzi* analysed (figure 1*c*; electronic supplementary material, table S5).

### 3.2. Clonally variant MASP expression

**Figure 2.**
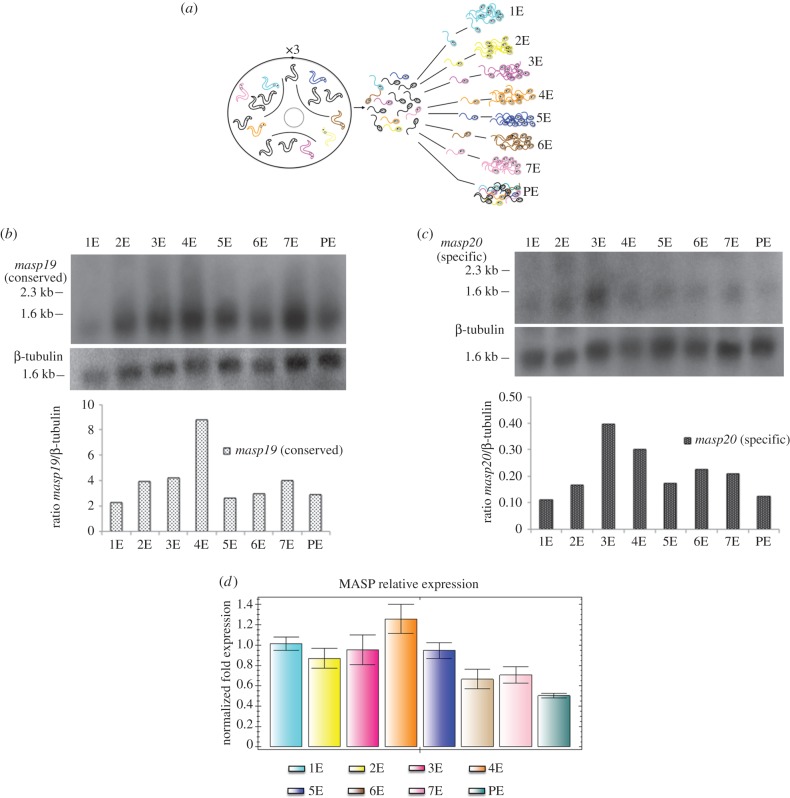
Study of the clonally variant MASP expression by northern blot and qRT-PCR. (*a*) Representation of the cloning process with three passages of the trypomastigtes through Vero cells and further cloning procedure to obtain the final clonal independent cell lines from the parental line. (*b,c*) Northern blot analysis of total RNA extracts from the epimastigotes forms of all the clones and the parental strain hybridized with radiolabelled *masp19* conserved probe (*b*) and *masp20* specific probe (*c*). β-tubulin was used as loading control in both northern blots. Below, bar graphs representing the ratio *masp19/*β-tubulin (*b*) and *masp20/*β-tubulin (*c*). (*d*) qRT-PCR analysis of the expression of the MASP family normalized with GAPDH. 1E–7E, name of each epimastigote clonal population; PE, epimastigote forms of the parental cell line.

To study the clonal heterogeneity in the expression of the MASP family, we analysed, at RNA and protein levels, the expression of seven clonally isogenic populations derived from single cells that were isolated from a parental culture of epimastigote cells belonging to the Pan4 strain (figure 2*a*).

At RNA level, both northern blot experiments revealed a clonally variant expression of the MASP transcripts. The hybridization with the *masp19* probe (sequence highly conserved in 23 of the 50 transcripts of the three-step MASP library, with percentage of identity ranging from 83 to 100%) produced a smear, suggesting the co-expression and detection of several MASP genes (figures 2*b* and 4). The ratio *masp19*/β-tubulin changed depending on the clone analysed. We observed an approximately fourfold-range of variation in expression between clones, with the highest signal corresponding to the clone4 and lowest to the clone1 (figure 2*b*). To find the variation of single MASP transcripts, we hybridized the membranes with the probe *masp20* (specific probe of the *masp20* transcript). As a whole, the signal was lower than that obtained with *masp19.* However, differences were also detected among the samples with again an approximately fourfold-range of variation between clones, with the clones 3 and 4 having the highest levels of relative expression. Again, the clone1 showed the lowest expression signal (figure 2*c*).

The analysis of the variability in the MASP expression of all isogenic parasite lines was also performed using two different PCR techniques: (i) qRT-PCR and (ii) three-step nested PCR resolved by capillary electrophoresis.

The qRT-PCR analysis allows us to obtain a global quantification of the MASP transcripts amplifying the conserved 5′-extreme region of this family on the epimastigote forms. These results also revealed clonal differences with an approximately 2.5-fold change variation in expression among the different isogenic parasite lines. The clone4 showed the highest MASP expression while the parental strain had the lowest levels (figure 2*d*).

**Figure 3.**
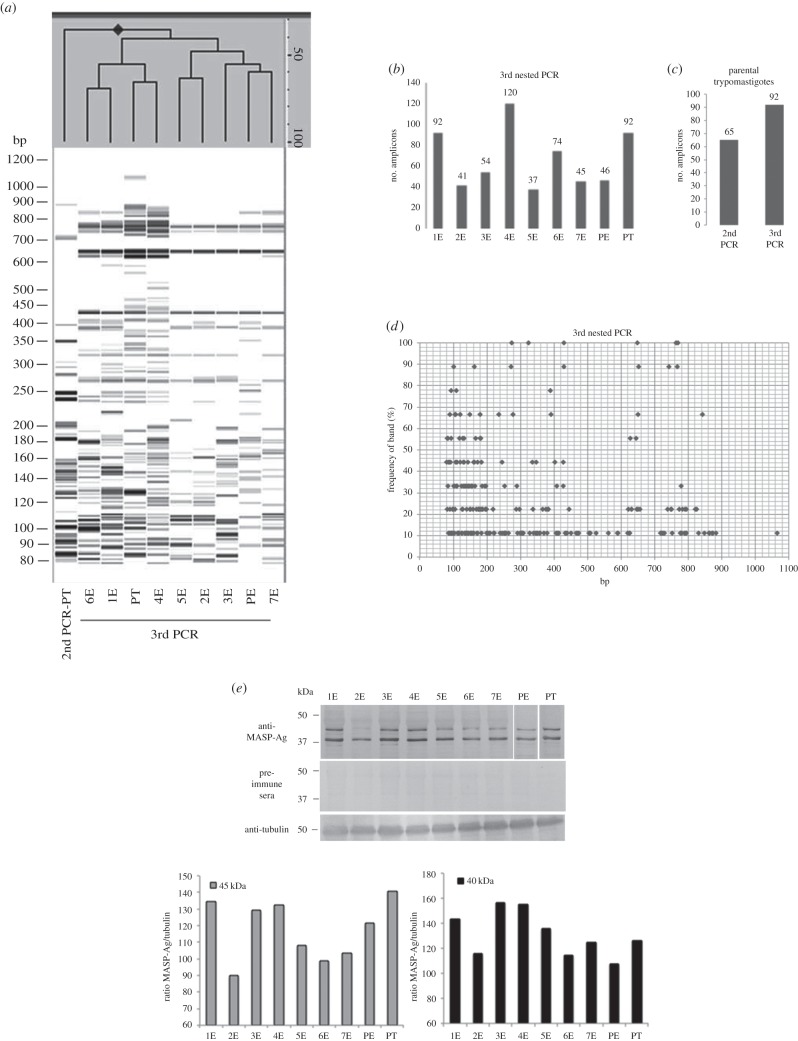
Analysis of MASP clonal expression by capillary electrophoresis and western blot. (*a*) *In silico* gel obtained by PCR fingerprinting analysing PCR products of three-step nested PCR for each isogenic line. On the top, a dendogram based on the similarity of the MASP amplicons pattern. The distances are measured at the right side of the dendogram with the percentage of similarity among the samples. (*b*) Bar graph representing the number of MASP amplicons obtained in the third step of the nested PCR for each clone and the parental strain. (*c*) Number of amplicons obtained from the second and the third steps of the nested PCR in the trypomastigotes of the parental cell line. (*d*) Frequency of bands according to their size amplified from the third nested PCR of the epimastigote stage for all the clones and from the epimastigote and trypomastigote forms of the parental cell line. (*e*) Western blot analysis of total protein extracts from the epimastigote forms of all the clones, and the epimastigote and trypomastigote stage of the parental strain using anti-MASP-Ag mouse antisera. Anti-tubulin sera were used as loading control and pre-immune sera as negative control. Below, bar graphs representing the ratio 45 kDa/tubulin and 40 kDa/tubulin. Second PCR and third PCR are the products of the second and third PCRs of the three-step nested PCR; 1E–7E, name of each epimastigote clonal population; PE, epimastigote forms of the parental cell line; PT, trypomastigote forms of the parental cell line.

Additionally, we resolved the products from the third nested PCR by capillary electrophoresis, elaborating a fingerprinting analysis. As a result, we obtained a high-resolution *in silico* gel where none of the cell lines analysed was transcriptionally identical. As was described by qRT-PCR, the clone4 showed the highest repertoire of bands in the *in silico* gel with 120 *masp* amplicons, a number even higher than that obtained by the Pan4 in its trypomastigote form with 92 bands (the stage where the highest MASP repertoire expression was expected) [28,30,31]. Meanwhile, the epimastigote form of the parental cell line showed one of the poorest profiles with 46 bands. The lowest number of *masp* amplicons was found in the clone5 profile, with 37 bands resolved (figure 3*a*,*b*). Another interesting result is the increase in the number of amplicons between the epimastigote stage, with the outlined 46 bands, and the trypomastigote stage of the parental cell line, with 92 bands. Among the 92 bands of the parental trypomastigote, 23 bands (25%) were specific to this stage and were not found in any other parasite line in the epimastigote stage. On top of the *in silico* gel, the dendogram based on the similarity of the pattern of the MASP amplicons (the absence/presence and intensity of the bands) allowed clustering of all the samples in different nodes. Here, the clone7 showed the most similar pattern to the parental strain in the epimastigote form (figure 3*a*). Furthermore, the frequency of appearance of the bands was determined according to their size for all the clones in the epimastigote stage and also for the parental line in the epimastigote and trypomastigote form. Unique bands were shown by 97 amplicons of a total of 601 (16%) and only six bands (1%) were present in all the samples. Thus, 83% of the bands were repeated in two to eight of the samples (figure 3*d*). Additionally, we also analysed the profile of the second semi-nested and third nested PCR product from the trypomastigote of Pan4. We obtained 65 and 92 bands, respectively, which confirm the increased sensitivity of the extra third step of PCR (figure 3*a*,*c*).

Finally, to analyse possible MASP expression heterogeneities at the protein level we used anti-MASP-Ag antibodies, which reacted with two epimastigote MASP proteins of 40 and 45 kDa in a clonally dependent manner. For the band of 45 kDa, the highest intensity of signal was shown in the trypomastigotes of the parental cell line while for the band of 40 kDa the clone3 and clone4 showed the highest intensities, even higher than the trypomastigotes of the parental line (figure 3*e*). On the other hand, the total protein profile of the same samples stained with Coomassie blue was not variable among the clones and the parental strain (electronic supplementary material, figure S7).

### 3.3. MASP RNA turnover assay

**Figure 4.**
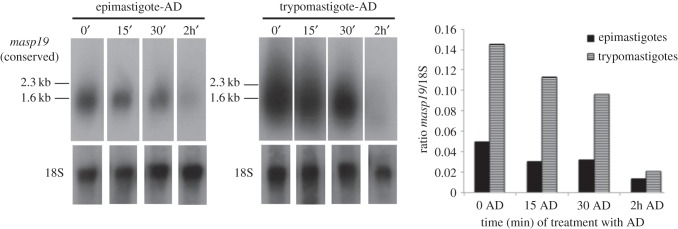
Study of the lifespan in MASP transcripts. Northern blot analysis of total RNA extracted from epimastigote and trypomastigote forms of Pan4, both treated with 10 µg ml^−1^ actinomycin D (AD) during different periods of time (0, 15, 30 min and 2 h) and hybridized with radiolabelled *masp19* conserved probe and with radiolabelled 18S probe as loading control. The bars represent the ratio *masp19/*18S.

The inhibitory effects of AD on transcription have been used to study the lifespan of MASP transcripts. Northern blot analysis shows the relative expression of the MASP genes from the epimastigote and trypomastigote detected by hybridization with the conserved *masp19* probe, which, as has been outlined, is potentially able to hybridize with several *masp* transcripts simultaneously (figure 4). The smear obtained mainly in trypomastigotes, but also in epimastigotes, suggests the ability of *masp19* to detect a wide repertoire of MASP transcripts expressed by Pan4*,* demonstrating the conserved character of the sequences detected. At time 0 h of the treatment with AD, we found a higher expression in the MASP recognized by the *masp19* probe in the trypomastigote forms, which was approximately threefold higher than in epimastigotes. At 2 h (end of the AD treatment) there is no significant difference in the RNA levels between both *T. cruzi* forms*.* Despite being difficult to accurately measure the intensity of that probe because of the smeary nature of its signal (especially in the case of 0, 15 and 30 min trypomastigote treatment), the lifespan seems to be the same for all the MASP RNAs detected in trypomastigotes and epimastigotes, with the hybridization signal almost lost after 2 h of AD treatment in both life-cycle stages (figure 4).

### 3.4. Phenotypic variations of clonally isogenic populations of cells

With the aim of detecting phenotypic differences, other aspects such as growth curves, the ability for metacyclogenesis and the metabolites secreted into the medium were analysed and compared among the isogenic cultures.

**Figure 5.**
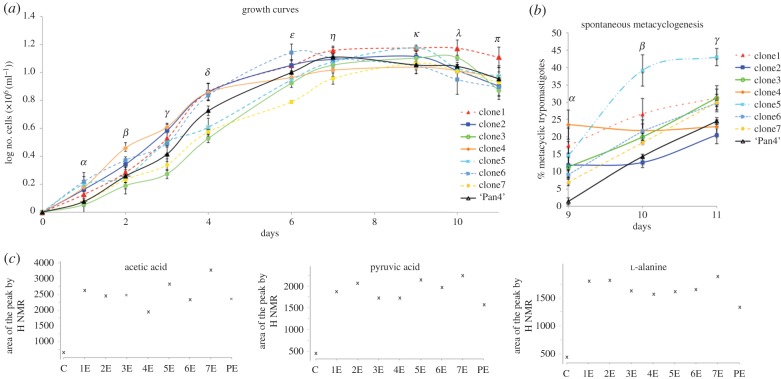
Phenotypic clonal variations among clones. (*a*) Growth curves of epimastigote cell lines. Letters above the time points mean that cell numbers among cell lines were significantly different (*p* ≤ 0.01) with the exception of: day 1, *α* (2vs4, 3vs7, 3vs8, 4vs5, 5vs6, 7vsPan4); day 2, *β* (5vs6); day 3, *γ* (5vs6); day 4, *δ* (1vs2, 1vs4, 2vs4); day 6, *ɛ* (1vs2); day 7, *η* (2vs6 and 6vsPan4); day 9, *κ* (1vs5, 2vs3, 4vs6, 4vs8, 6vs7, 6vs8, 7vsPan4); day 10, *λ* (2vs4, 2vs5, 2vs7, 4vs5, 4vs7, 5vsPan4); day11,*π* (2vs6, 4vs5, 4vsPan4 and 5vsPan4). The bars represent the standard error of the mean of the three independent biological replicates. (*b*) Spontaneous metacyclogenesis yields at day 9, 10 and 11 of the culture. Letters above the time points mean that cell numbers among cell lines were significantly different (*p* ≤ 0.01) with the exception of: day 9, *α* (2vs3); day 10, *β* (4vs6); day 11, *γ* (1vs3, 6vs7). (*c*) Area under the peak obtained from the H NMR spectrums for acetic acid, pyruvic acid and l-alanine produced and excreted during the epimastigote cultures of the isogenic lines. (*c*) RPMI with 10% IFSC (blank); 1E–7E, name of each epimastigote clonal population; PE, epimastigote forms of the parental cell line.

In the growth curve analyses, despite describing normal growth kinetics as a general trend, each clone manifested to a greater or lesser extent significant differences from the first day of growth (figure 5*a*). The first clone to reach the stationary phase was clone7 at the sixth day of growth, one day earlier than the parental strain and two days earlier than the rest of the clones. On the other hand, the highest cellular densities were observed in clones 1 and 5, both with 1.175 × 10^7^ cells. Once all the cultures reached the stationary phase, each of them showed a different decreasing speed, ending the counts in a similar parasite density. Only clone1 presented a significantly higher number of epimastigotes at the 11th day (figure 5*a*).

Regarding the spontaneous metacyclogenesis, significantly different dynamics in metacyclic transformation were detected among the cell lines. Clone5 showed the best ability to differentiate into metacyclic forms with more than the 40% of the cells in metacylic stage at the 11th day of culture. In contrast, clone2 presented the lowest metacylogenesis activity with 21% while Pan4 had 25% of metacyclic forms in culture at 11th day (figure 5*b*).

With respect to the metabolites produced and excreted during the epimastigote cultures for each isogenic parasite line, we identified differences in the signal intensities of the proton spectrums obtained from acetic acid, l-alanine and pyruvic acid. The highest differences were found for acetic acid secretion with approximately 1.5-fold changes in the production of this metabolite between clone4 (lowest) and clone7 (highest). Moreover, in the l-alanine and pyruvic acid spectrums, clone7 also showed the highest yields of secreted metabolites, contrary to the parental cell line, which showed the lowest levels for l-alanine and pyruvic acid production (figure 5*c*).

## 4. Discussion

Clonal variations have been postulated as an intrinsic property of protozoan populations in order to facilitate the adaptation to different environments or conditions during the life cycle of the parasite. These variations have been related not only to individual genes but also to gene families involved in host–parasite interactions [16,23,50–52]. Owing to its abundant presence in the genome, the surface location and the ability to be secreted by the parasite, the multigene MASP family of *T. cruzi* is believed to be a key factor in the success of cell infection and the survival of the protozoan in the mammalian host [25,28,30]. However, MASP expression is not only present in the mammalian forms of *T. cruzi* (bloodstream trypomastigote and amastigotes) but also in the epimastigote and metacyclic stages present in the insect vector [19,30], having no orthologues in any other species [26].

Despite the importance of this protein family for the biology of this trypanosomatid, the evidence for clonality and, hence, variation among *T. cruzi* cell populations remains poorly characterized. In this study, we have analysed the variability in MASP expression and its protein synthesis as well as other phenotypic traits in *T. cruzi* after cloning the TcI Pan4 strain of the parasite in seven different clones.

With this aim, the following techniques have been applied: (i) northern blot; (ii) qRT-PCR; (iii) three-step nested PCRs resolved by capillary electrophoresis; and (iv) western blot, to provide experimental evidence that clonally isogenic populations of epimastigotes are highly heterogeneous.

The capillary electrophoresis of the amplicons has been shown as a highly resolutive method for the high definition separation of the three-step nested PCR products ranging in length from 80 to 1100 bp, and thus allowing for the identification of specific differences in the MASP expression among the clonal isolates and the parental cell line (figure 3*a*). Furthermore, the addition of a third reaction to the two semi-nested PCRs described by dos Santos *et al.* showed an increase of 29% in the number of MASP amplicons of the trypomastigotes of Pan4 (figure 3*c*). Using this methodology, we have confirmed the huge repertoire of MASP amplicons expressed not only in trypomastigotes but also in the epimastigote stage [28–30], and more importantly, we have shown that none of the clonal isogenic populations isolated was transcriptionally identical (figure 3*a*,*b*). In fact, qRT-PCR, northern blot and western blot analysis also confirmed the existence of important clonal variations in the MASP expression at the RNA and protein levels among the epimastigote isogenic clones and the parental line. Moreover, contrary to expectations, the parental line Pan4 tends to show the lowest values of MASP diversity of bands when compared with the clones, whereas clone4 is characterized by its high levels of MASP expression at RNA and protein level (figures 2–4).

We have also used the three-step nested PCR to obtain a highly specific library of MASP expressed transcripts (named three-step MASP library). The three-step MASP library, plus the smear obtained with a conserved probe (*masp19*) in the northern blots assays (figures 2*b*,*c* and 4) and the capillary electrophoresis (figure 3*a–d*) confirm the great MASP co-expression that occurs at RNA level in the trypomastigote and epimastigote stages of the parental line and in the clonal isogenic populations of epimastigotes. We also showed that expression levels of the transcripts, recognized by the *masp19* probe, were higher in trypomastigotes than epimastigotes; however, both life-cycle stages showed a similar MASP RNA lifespan, close to 2 h after the AD treatment (figure 4). This similar regulation of the MASP mRNAs is in accordance with the extremely high degree of conservation of the MASP 3′UTRs [28] and hence *cis*-regulatory sequences [31]. Thus, differences in expression and patterns between the different life-cycle stages might be explained by other differences such as levels of *trans*-acting factors (i.e. RNA-binding proteins and eukaryotic initiation translation factors), different codon usage and tRNA pools available for those sequences, as well as by the genomic plasticity shown by the replicative forms of the parasite (epimastigote and amastigote) [32,33,53,54].

Despite the great variability of the MASP family, we have found by capillary electrophoresis the presence of six bands with a conserved size among the epimastigote clones and the parental strain in the epimastigote and trypomastigote stages (figure 3*a*,*d*), suggesting that there is a trend of positive selection, which could favour the presence of some MASPs. In this regard, the main pairwise distances obtained by comparing the genes of our three-step MASP library to the *T. cruzi* genome of other strains in the hierarchical cluster analyses allowed us to find a distribution according to their phylogenetic origin for sequences codifying for MASP genes (figure 1*b*). In this analysis, we also defined two groups (slow and fast evolving sequences) in gene and pseudogene datasets (figure 1*b*,*c*). Nevertheless, the *masp10.ps* was an exception and was not included in the two main groups of the pseudogene hierarchical cluster distribution (figure 1*c*). Because of the high mean pairwise distances showed for *masp10.ps* with respect to the strains Silvio X10/1, Dmc28 and Marinkellei, this pseudogene is the fastest evolving member of the library. However, *masp10.ps* showed high percentages of identity in respect to different MASPs in the genome of CL Brener, in the three strains of our preliminary MASP expression libraries and in three of the seven MASP expression libraries performed by dos Santos *et al.* [29] (figure 1*c*,*d*). These data suggest that a proportion of the MASP transcriptome is highly conserved not only among the life-cycle stages but also among different strains. In fact, we can hypothesize that MASP pseudogenes could also conserve their sequences, being positively selected, to play some biological function probably acting at RNA level as regulators of gene expression. For instance, it has been found that the pseudogenes are a source of antisense RNAs in *Lymnaea stagnalis* where, for instance, the expression of NO synthase (NOS) is regulated by the co-transcription of the pseudo-NOS transcript which forms a long RNA duplex with NOS and thus selectively suppresses the expression of the enzyme depending on the presence of the pseudogene [55]. Additionally, the constant remodelling and differential MASP expression might serve as a source of novel specializations on the surface of the parasite that could favour and fix the presence of some MASP proteins rather than others, as occurs in its close relative *T. brucei* where the transferrin receptor, haptoglobin–haemoglobin receptor (HpHbR) or serum resistance associated (SRA) have evolved from variant surface glycoprotein (VSG) gene family [56–59].

Moreover, the results obtained by aligning the isolated expressed sequences with very similar size (maximum 3 bp of difference) from the three-step MASP library and the experimental evidence that we have provided in this article prompt us to suggest that the variation in sequence (SNPs) and the variation in size could be a result of the MASP clonal expression structure of *T. cruzi* populations. This MASP clonal variation may be sustained by two evolutional strategies: by adaptive plasticity mechanisms, involving environmental sensors which respond to external changes, or by a stochastic clonal expression strategy in which the cells are constantly and randomly replacing their repertoire. In this regard, the adaptation of cells to the culture after the cloning procedure and the unique features of each isolated clone might determine the differences found in MASP expression. Alternatively, it could also be possible that we selected for the better adapted cells which are also constantly changing, so our results provide a snapshot of the transcriptional diversity in a given time after several replications. Clonal variability in transcription was also reflected by differences in phenotypic traits such as growth curve, spontaneous metacyclogenesis or the levels of some catabolites secreted into the medium (figure 5). At the logarithmic phase of the growth curve, the parasites did not reach the same numbers and also had not secreted the same yields of acetic acid, pyruvic acid and l-alanine, which are end products of glucose catabolism [60], showing again heterogeneities among the isogenic lines. During exponential growth, cells consume sugar and dissimilate acetate. Acetogenesis, the excretion of acetate into the environment, results from the need to regenerate the NAD+ consumed by glycolysis and to recycle the coenzyme A (CoASH) required to convert pyruvate to acetyl-CoA, the central point of the metabolism [61]. Acetate is known to be an essential product for lipid biosynthesis, thus we can assume that the high amount of GPI-anchored proteins which are clonally expressed by the epimastigote populations could also be reflected in the different production of fatty acids by those cell populations [62,63]. Although we were not able to find a complete biological connection between clonal heterogeneities in MASP expression and the phenotypic parameters analysed, it is remarkable that the parental cell line always shows the lowest MASP transcriptional levels, spontaneous metacyclogenesis and production of metabolites.

Our studies of phenotypic mosaicism were focused on the epimastigote stage, the main parasite form in the triatomine which is present from the stomach (dilated anterior part of the midgut) to the rectum of the insect [19]. In this journey, the epimastigotes are responsible for the replicative process (in the small intestine) and for the transformation into metacyclic trypomastigotes (in the rectum) [64]. To fulfil these functions and establish the infection, the epimastigotes have to overcome biochemical and physiological factors present in the insect vector gut. These include digestive enzymes (cathepsins, lysozmes and cysteine proteases, etc.), pH and nutritional changes and components of the vector humoral immune system [19,65]. Additionally, the success of the infection is dependent on the attachment of the parasite to the gut epithelial surfaces [19]. Diversity confers fitness in a changing population, so MASP clonally variant expression at the epimastigote stage could provide a great repertoire of these molecules which may function to thwart the insect's immune response, participate in the parasite attachment interactions with the gut epithelium surfaces or even confer protection against the digestive enzymes of the insect vector [19,31,66].

The absence of transcriptional regulation in kinetoplastid parasites also limits the capacity of those parasites to mount a direct response to external changes; thus, pre-existing pools of heterogenic cells will favour the survival of the cells in constantly changing niches, although whether or not this is a pre-existing characteristic or a consequence of the environment changes is still unknown.

This study attempts, for the first time, to provide a comprehensive characterization of the MASP expression heterogeneity among clonal isogenic populations of *T. cruzi*. However, single-cell approaches will be necessary to clarify whether a single parasite is able to co-express different members of the MASP family simultaneously or, on the contrary, the co-expression found is a result of the pool of cells that constitutes a population where every parasite expresses a single MASP gene.

## Supplementary Material

Supplementary figures and tables
