# Supplementary material for: Transcriptional and phenotypical heterogeneity of Trypanosoma cruzi cell populations
Source: Open Biol. 2015 Dec 16;5(12):150190. doi: 10.1098/rsob.150190 (PMC4703061; doi:10.1098/rsob.150190)
Supplement: Supplementary figures and tables [file rsob150190supp1.pdf]

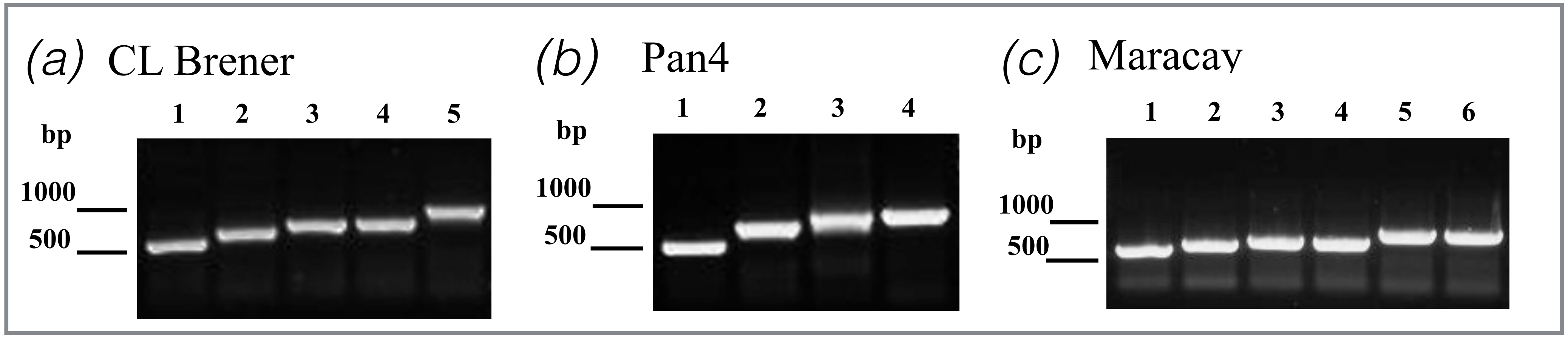

Figure S1. Inter-strain MASP expression library in *T. cruzi* . Agarose gel (1%) of the amplicons in: (*a*) CL Brener strain. 1- masp1CL; 2- masp2Ps.1CL; 3- masp3CL; 4- masp4Ps.2CL; 5- masp5Ps.3CL. (*b*) Pan4 strain. 1- masp1Ps.1Pan4; 2- masp2Ps.2Pan4; 3- masp3Ps3Pan4; 4- masp4Pan4. (*c*) Maracay strain. 1- masp1Mar; 2- masp2Mar; 3- masp3Mar; 4- masp4Mar; 5- masp5Ps.1Mar; 6- masp6Mar.

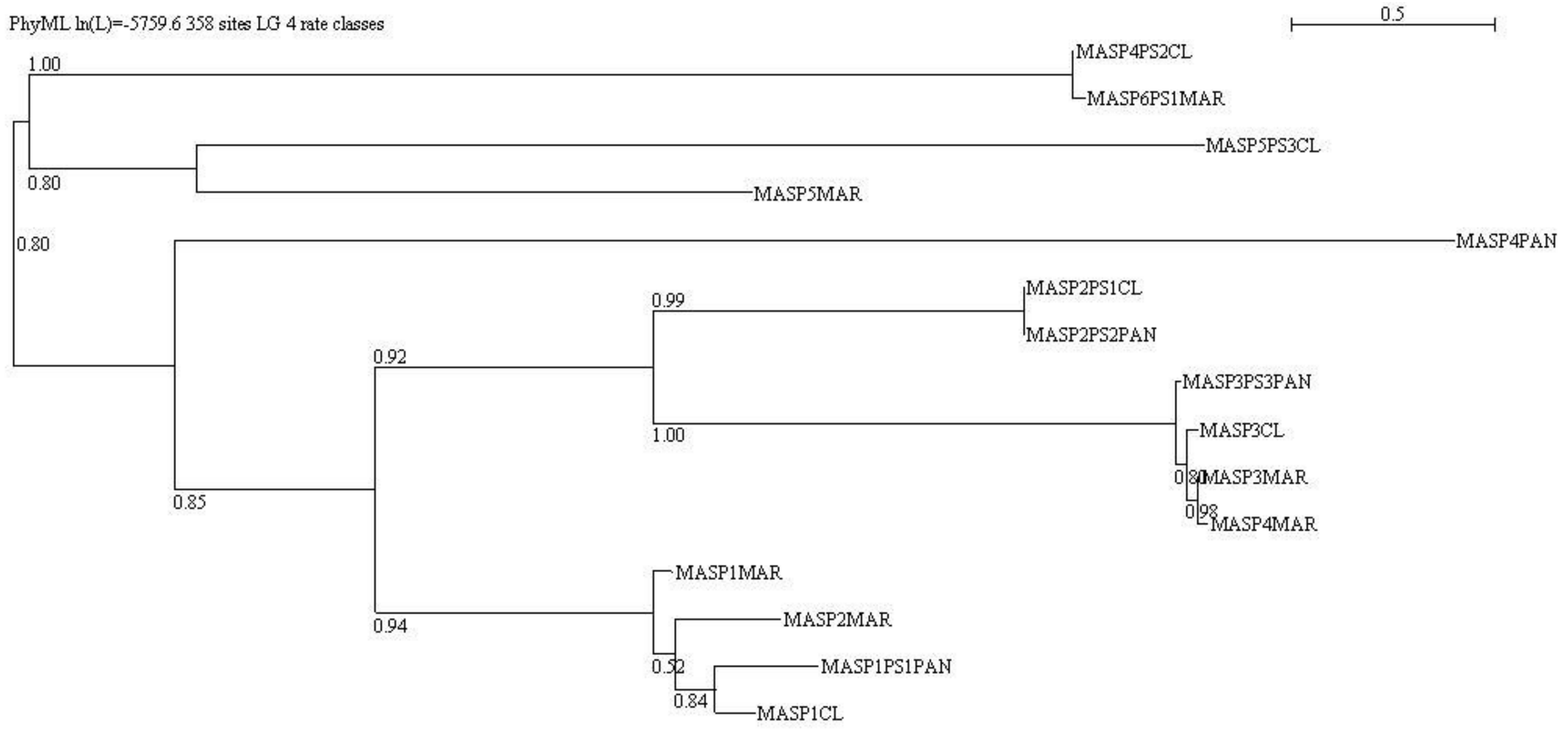

Figure S2. Phylogenetic reconstruction of MASP expressed (pseudo)genes found in the preliminary inter-strain expression libraries. The evolutionary distances were computed in Mega 6.06 software (Tamura et al. 2013) using the Maximum Composite Likelihood phylogeny test (Tamura, Nei, Kumar 2004) and the Tamura-Nei model. PS: pseudogen, MAR:maracay strain, CL: CL-Brener strain.

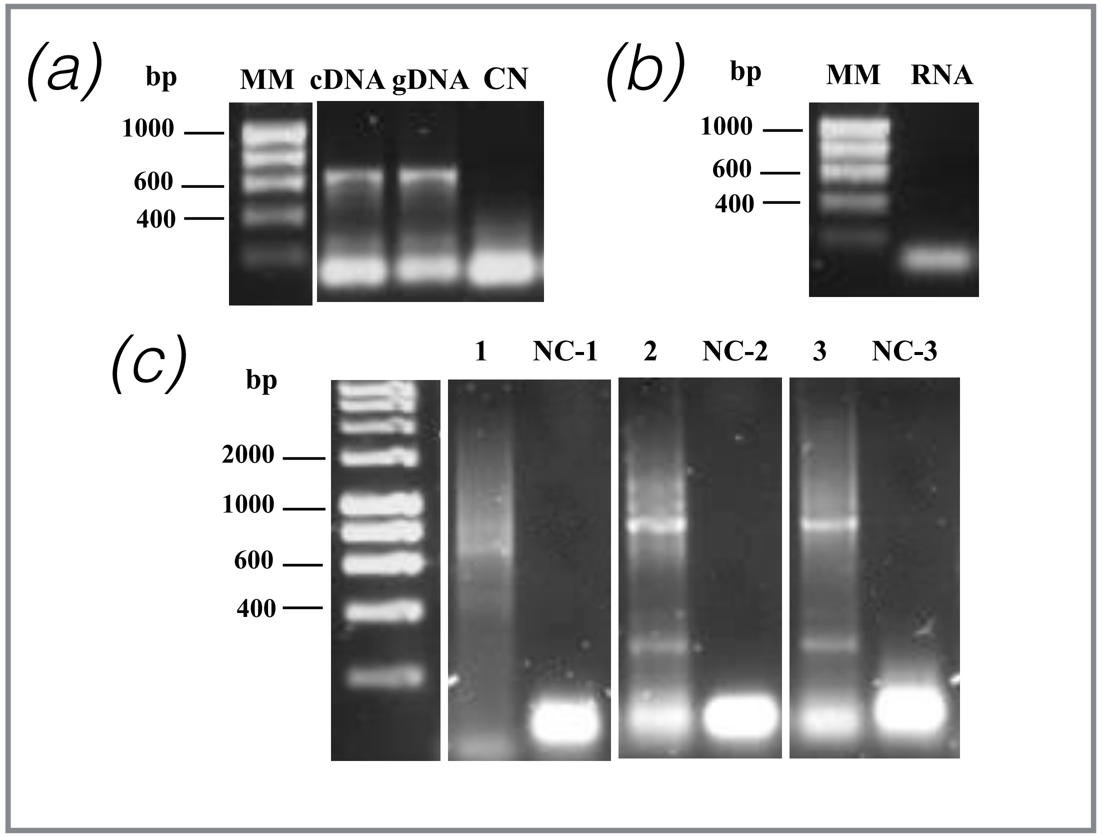

Figure S3. Construction of the MASP expression library from trypomastigotes of the Pan4 strain by 3-step nested PCR: agarose electrophoresis gels (1%) for RNA- cDNA (quality controls) and for the 3-step nested PCR for MASP amplification. (*a*) PCR for AHADH2 gene using the cDNA, gDNA and water as negative control. (*b*) Negative control PCR for AHADH2 gene using total RNA as template. (*c*) Three steps of the nested PCR reaction: 1- first PCR reaction for MASP family using cDNA as template- with primers SL and 3′UTR1. 2- second PCR reaction for MASP family using the first PCR product as template- with primers SL and 3′UTR2. 3- third PCR reaction for the amplification of MASP specific transcripts using the second PCR product as template- with primers MASP N-term and MASP C-term. gDNA: PCR using 10 ng *T. cruzi* genomic DNA as template; NC-1- NC-2- NC-3: negative control of PCR reaction- with no DNA template for the first, the second and the third PCR reactions respectively.

(a)

(b)

(c)

(d)

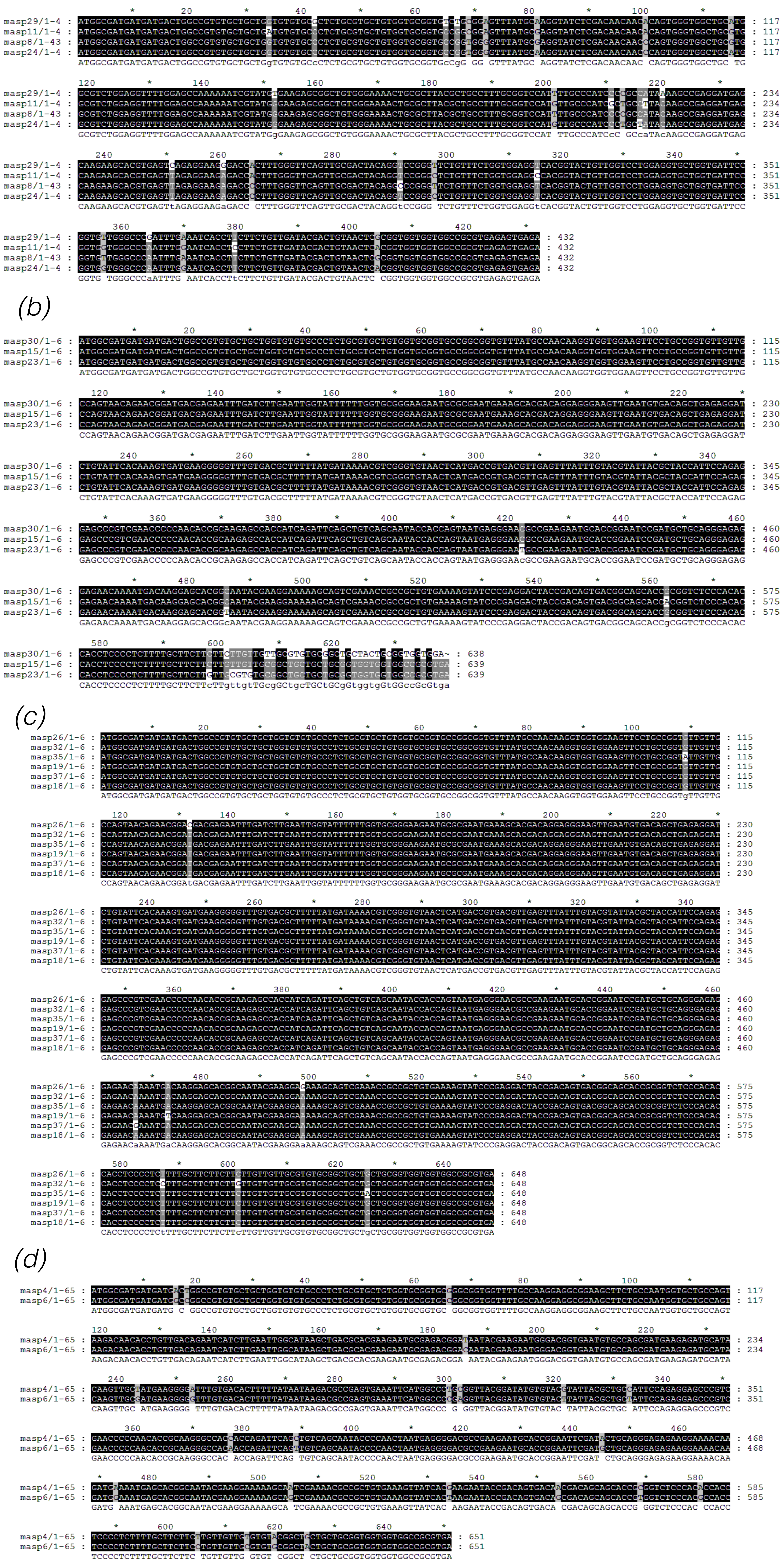

Figure S4. SNP's analysis by multiple alignment among different MASP sequences from the 3-step MASP library with a length of 432bp (*a*), 638 and 639bp (*b*), 648bp (*c*), 651bp (*d*). Complete homology among the sequences is represented with black areas whereas nucleotides in grey represent areas with less than 80% of homology.

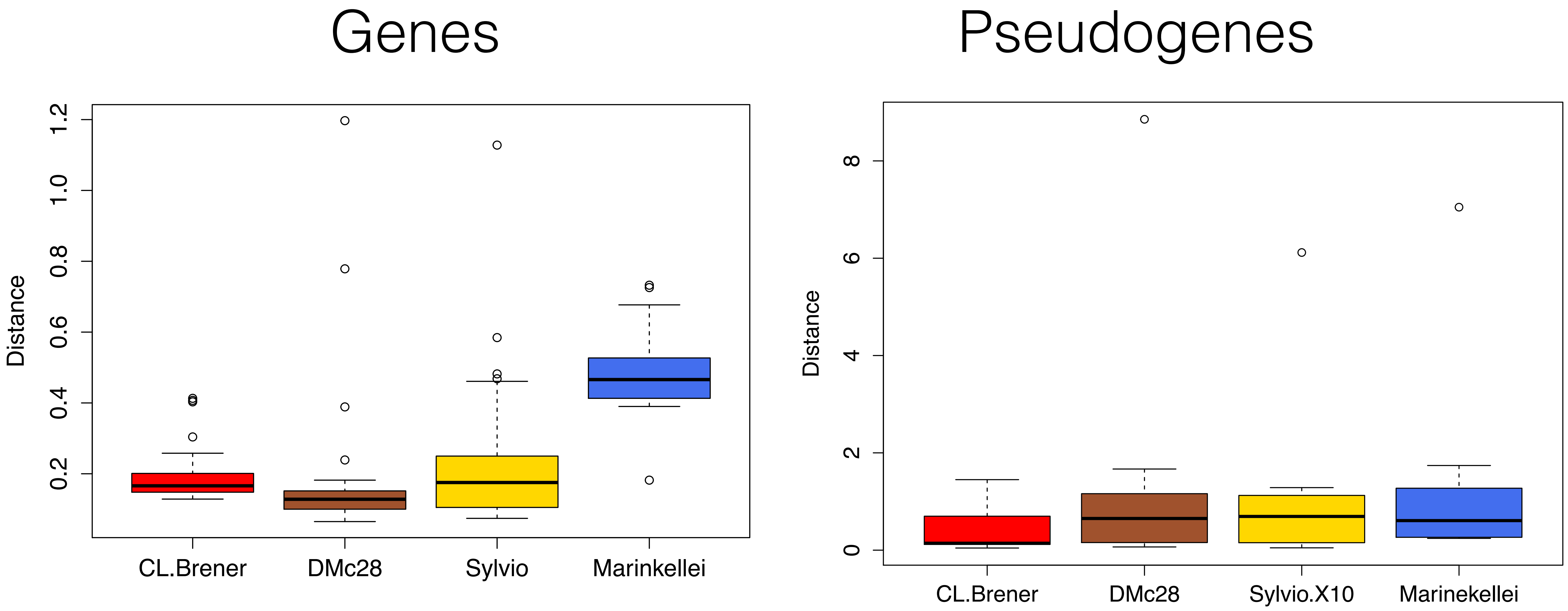

Figure S5. Boxplots representing the pairwise distances between the 3-step MASP library and the ortholog sequences of CL Brener, Dm28c, Sylvio X10/1 and Marinkellei. The boxplots show the distribution of the mean pairwise distances values for the ortholog sequences among the different strains.

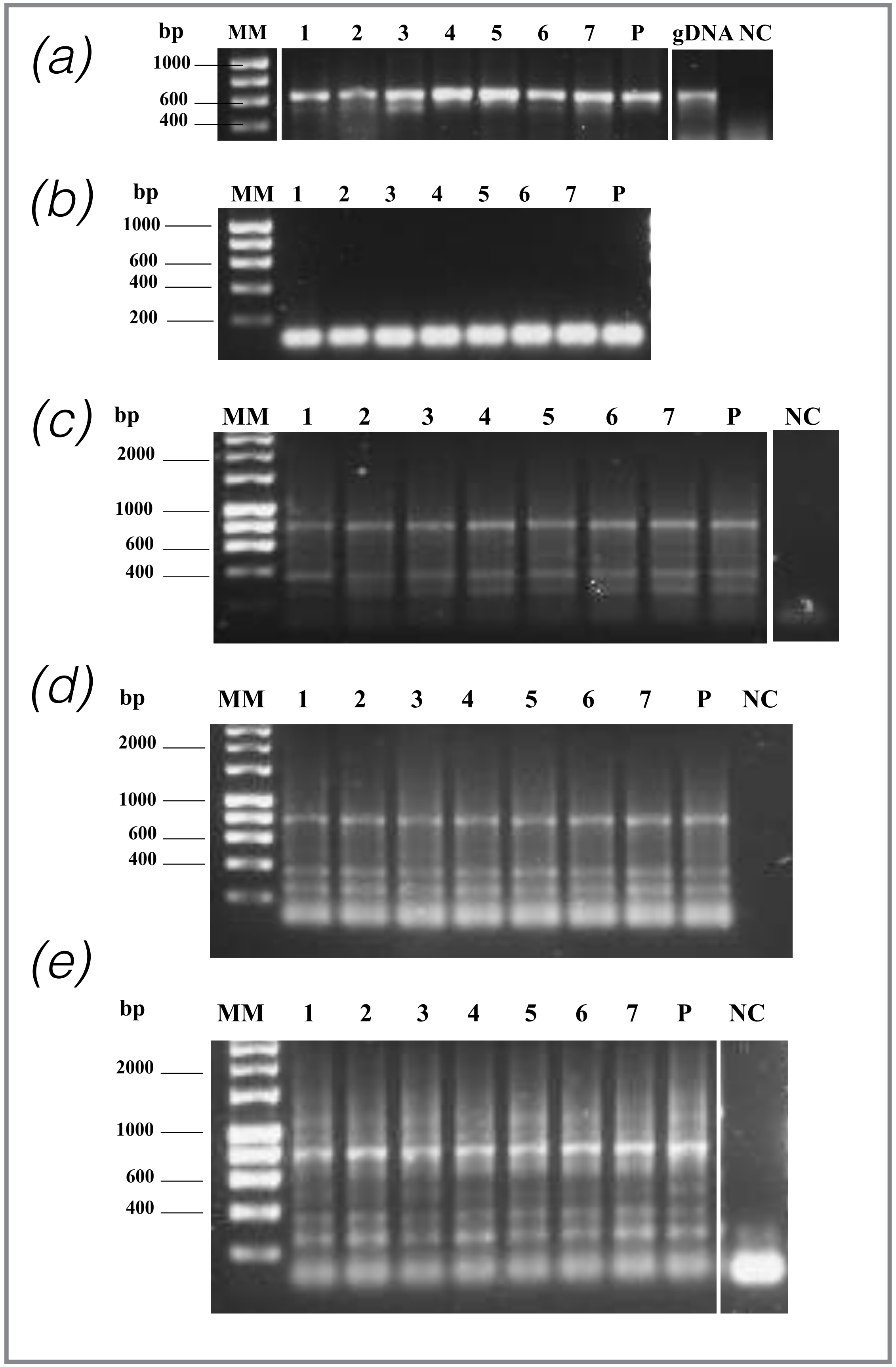

Figure S6. 3-step nested PCR for clonal MASP expression analysis in the epimastigote form: agarose electrophorese gels (1%) for RNA- cDNA (quality controls) and for the 3-step nested PCR for MASP amplification. (*a*) PCR for AHADH2 gene using the cDNA as template. (*b*) Negative Control PCR for AHADH2 gene using total each RNA as template. (*c*) First PCR reaction for MASP family using each cDNA as template- with primers SL and 3′UTR1. (*d*) Second PCR reaction for MASP family using each first PCR product as template- with primers SL and 3′UTR2. (*e*) Third PCR reaction for MASP family using each second PCR product as template- with primers MASP N-term and MASP C-term. [1-7]- name of each clone; P- parental strain (Pan4).

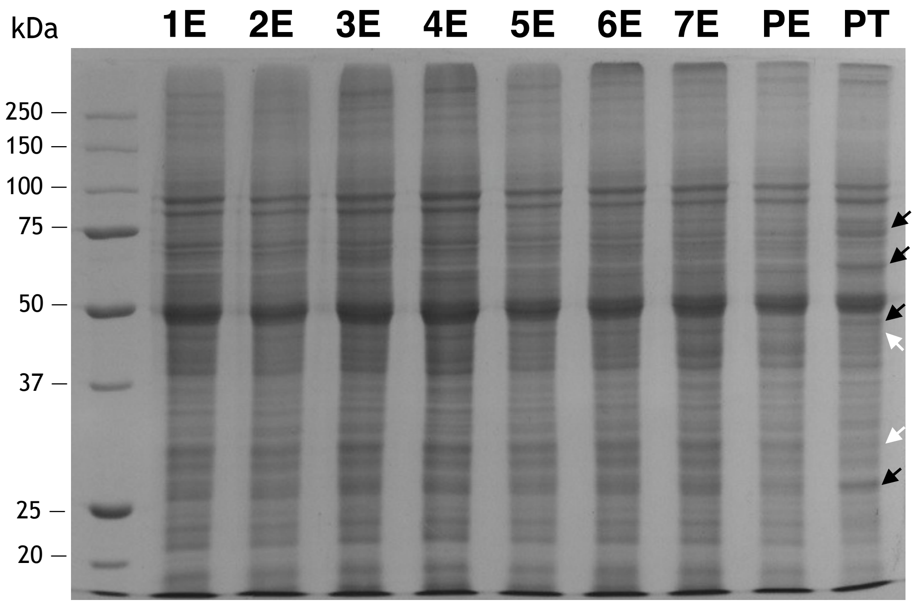

Figure S7. Protein extracts from the isogenic parasite populations electrophoresed and stained with Comassie brilliant blue. The black arrows indicate the bands overexpressed while the white arrows indicate those which are underexpressed in PT respect PE. [1E-7E], name of each epimastigote clonal population; PE, epimastigote forms of the parental cell line; PT, trypomastigote forms of the parental cell line.

Table S1-Inter-strain masp expression library

CL Brener			Pan4			Maracay		
**Clone**	**Lenght bp;aa**	**ENA ID**	**Clone**	**Lenght bp;aa**	**ENA ID**	**Clone**	**Lenght bp;aa**	**ENA ID**
masp1CL	522; 184	HE600128	***masp1Ps.1Pan4***	521; 141 (Stop)	HE600133	***masp1Mar***	552; 184	HE600137
masp2Ps.1CL	619; 64 (Stop)	HE600129	***masp2Ps.2Pan4***	619; 64 (Stop)	HE600134	***masp2Mar***	660; 220	HE600138
masp3CL	771; 257	HE600130	***masp3Ps3Pan4***	765; 130 (Stop)	HE600135	***masp3Mar***	756; 252	HE600139
masp4Ps.2CL	977; 31 (Stop)	HE600131	***masp4Pan4***	909; 303	HE600136	***masp4Mar***	759; 253	HE600140
masp5Ps.3CL	1065; 133 (Stop)	HE600132				***masp5Ps.1Mar***	978; 56 (Stop)	HE600142
						masp6Mar	996; 326	HE600141

Table S2- Sequencing results of the 3-step Pan4 library

Nº clones sequenced	Valid sequences	%Valid sequences	Masp hits*	%Masp hits	Masp complete gene sequence	Masp pseudogene sequence	Unique Masp complete gene	Unique Masp pseudogene	% Unique Masp complete gene	% Unique Masp pseudogene
62	58	93,5	57	98,3	45	12	37	12	82,2	100,0

Table S3- List of MASP genes from the 3-step MASP library and their respective orthologs in the different strains

GENES	pb	aa	Genbank ID	CL Brener				Dm28c				Sylvio X10/1				Marinkellei strain B7			
				Annotation	Best Hit	Score	E-value	Annotation	Best Hit	Score	E-value	Annotation	Best Hit	Score	E-value	Annotation	Best Hit	Score	E-value
masp1	648	219	KT222275	MASP	TcCLB.505025.130	340	2E-118	MASP	TCDM_10876	342	1E-118	MASP	TCSYLVIO_009410	322	1E-110	MASP	Tc_MARK_5257	216	1E-69
masp2	648	219	KT222276	MASP	TcCLB.505025.130	343	2E-119	MASP	TCDM_10876	344	2E-119	MASP	TCSYLVIO_009410	325	1E-111	MASP	Tc_MARK_5257	218	1E-70
masp3	648	220	KT222277	MASP	TcCLB.508871.60	347	7E-121	MASP	TCDM_10324	356	2E-124	MASP	TCSYLVIO_009410	330	1E-113	MASP	Tc_MARK_5257	204	7E-65
masp4	651	221	KT222278	MASP	TcCLB.508871.60	358	1E-125	MASP	TCDM_10220	358	7E-125	MASP	TCSYLVIO_009410	317	1E-108	MASP	Tc_MARK_5257	215	2E-69
masp5	392	136	KT222279	MASP	TcCLB.510629.350	189	1E-58	MASP	TCDM_10436	186	2E-58	MASP	TCSYLVIO_002084	154	5E-46	MASP	Tc_MARK_9088	133	5E-38
masp6	651	216	KT222280	MASP	TcCLB.508871.60	351	4E-123	MASP	TCDM_10220	356	6E-124	MASP	TCSYLVIO_009410	313	5E-107	MASP	Tc_MARK_5257	209	5E-67
masp7	648	220	KT222281	MASP	TcCLB.508871.60	347	7E-121	MASP	TCDM_10324	356	2E-124	MASP	TCSYLVIO_009410	330	1E-113	MASP	Tc_MARK_5257	204	7E-65
masp8	432	149	KT222282	MASP	TcCLB.511255.280	201	1E-62	MASP	TCDM_09622	281	1E-93	MASP	TCSYLVIO_009298	137	4E-38	MASP	Tc_MARK_8983	120	5E-32
masp9	852	288	KT222283	MASP	TcCLB.504239.420	368	4E-127	MASP	TCDM_10569	484	8E-173	MASP	TCSYLVIO_002050	249	5E-81	MASP	Tc_MARK_75	215	7E-67
masp10	780	259	KT222284	MASP	TcCLB.506001.14	276	2E-91	MASP	TCDM_10436	330	7E-113	MASP	TCSYLVIO_002084	154	5E-44	MASP	Tc_MARK_10309	177	3E-52
masp11	432	149	KT222285	MASP	TcCLB.511255.280	204	8E-64	MASP	TCDM_09709	228	5E-73	MASP	TCSYLVIO_009298	137	4E-38	MASP	Tc_MARK_8983	121	3E-32
masp12	201	71	KT222286	MASP	TcCLB.510629.70	122	6E-34	MASP	TCDM_10436	114	5E-32	MASP	TCSYLVIO_002084	115	4E-32	MASP	Tc_MARK_6934	100	2E-26
masp13	285	94	KT222287	MASP	TcCLB.511173.460	141	2E-41	MASP	TCDM_12580	63.5	1E-12	MASP	TCSYLVIO_006684	62.4	3E-12	MASP	Tc_MARK_5647	69.3	3E-15
masp14	645	214	KT222288	MASP	TcCLB.506757.50	326	6E-113	MASP	TCDM_08758	321	2E-110	MASP	TCSYLVIO_008640	324	3E-112	MASP	Tc_MARK_5257	235	3E-77
masp15	639	217	KT222289	MASP	TcCLB.506757.50	327	3E-113	MASP	TCDM_08758	324	6E-112	MASP	TCSYLVIO_008640	325	2E-112	MASP	Tc_MARK_5257	236	2E-78
masp16	652	223	KT222290	MASP	TcCLB.506757.50	322	2E-111	MASP	TCDM_08758	316	1E-108	MASP	TCSYLVIO_008640	318	6E-110	MASP	Tc_MARK_5257	232	2E-76
masp17	927	308	KT222291	MASP	TcCLB.510013.290	278	4E-91	MASP	TCDM_08850	404	2E-140	MASP	TCSYLVIO_006646	406	2E-141	MASP	Tc_MARK_6490	148	3E-40
masp18	648	215	KT222292	MASP	TcCLB.506757.50	329	5E-114	MASP	TCDM_08758	322	3E-111	MASP	TCSYLVIO_008640	326	7E-113	MASP	Tc_MARK_5257	238	2E-78
masp19	648	215	KT222293	MASP	TcCLB.506757.50	328	2E-113	MASP	TCDM_08758	319	6E-110	MASP	TCSYLVIO_008640	323	1E-111	MASP	Tc_MARK_5257	237	6E-78
masp20	798	265	KT222294	MASP pseudogene	TcCLB.504261.100	234	2E-75	MASP	TCDM_10163	285	6E-95	MASP	TCSYLVIO_005163	264	9E-87	MASP	Tc_MARK_5457	104	8E-26
masp21	945	319	KT222295	MASP	TcCLB.511797.167	294	8E-97	MASP	TCDM_10436	301	1E-100	MASP	TCSYLVIO_002084	214	2E-66	MASP	Tc_MARK_10309	185	1E-54
masp22	648	215	KT222296	MASP	TcCLB.508871.60	347	7E-121	MASP	TCDM_10324	356	2E-124	MASP	TCSYLVIO_009410	330	1E-113	MASP	Tc_MARK_5257	204	7E-65
masp23	639	212	KT222297	MASP	TcCLB.506757.50	327	4E-113	MASP	TCDM_08758	321	1E-110	MASP	TCSYLVIO_008640	324	3E-112	MASP	Tc_MARK_5257	236	1E-77
masp24	432	144	KT222298	MASP	TcCLB.511255.280	204	1E-63	MASP	TCDM_10606	283	3E-94	MASP	TCSYLVIO_009298	138	2E-38	MASP	Tc_MARK_8983	119	2E-31
masp25	648	221	KT222299	MASP	TcCLB.506757.50	320	3E-110	MASP	TCDM_08758	310	3E-106	MASP	TCSYLVIO_008640	306	5E-105	MASP	Tc_MARK_5257	231	2E-75
masp26	648	215	KT222300	MASP	TcCLB.506757.50	329	5E-114	MASP	TCDM_08758	322	3E-111	MASP	TCSYLVIO_008640	326	7E-113	MASP	Tc_MARK_5257	238	4E-79
masp27	645	220	KT222301	MASP	TcCLB.508873.210	318	4E-109	MASP	TCDM_08758	329	6E-114	MASP	TCSYLVIO_008640	347	5E-121	MASP	Tc_MARK_5257	237	5E-78
masp28	612	204	KT222302	MASP	TcCLB.510961.10	248	1E-79	MASP	TCDM_11069	54.7	1E-08	MASP	TCSYLVIO_009300	53.1	3E-08	MASP	Tc_MARK_3973	211	2E-65
masp29	432	144	KT222303	MASP	TcCLB.511255.280	199	8E-62	MASP	TCDM_09709	239	4E-77	MASP	TCSYLVIO_009298	136	7E-38	MASP	Tc_MARK_8983	115	5E-30
masp30	638	219	KT222304	MASP	TcCLB.506757.50	328	9E-114	MASP	TCDM_08758	322	5E-111	MASP	TCSYLVIO_008640	326	8E-113	MASP	Tc_MARK_5257	238	3E-78
masp31	648	215	KT222305	MASP	TcCLB.508873.210	312	8E-107	MASP	TCDM_08758	315	2E-108	MASP	TCSYLVIO_008640	327	4E-113	MASP	Tc_MARK_5257	229	5E-75
masp32	648	215	KT222306	MASP	TcCLB.506757.50	329	7E-114	MASP	TCDM_08758	322	5E-111	MASP	TCSYLVIO_008640	326	7E-113	MASP	Tc_MARK_5257	238	3E-78
masp33	651	216	KT222307	MASP	TcCLB.506757.50	303	1E-103	MASP	TCDM_08758	297	4E-101	MASP	TCSYLVIO_008640	307	1E-105	MASP	Tc_MARK_5257	232	5E-76
masp34	410	137	KT222308	MASP	TcCLB.510629.350	189	1E-58	MASP	TCDM_10436	186	1E-58	MASP	TCSYLVIO_002084	154	5E-46	MASP	Tc_MARK_9088	133	5E-38
masp35	648	215	KT222309	MASP	TcCLB.506757.50	328	9E-114	MASP	TCDM_08758	322	6E-111	MASP	TCSYLVIO_008640	326	7E-113	MASP	Tc_MARK_5257	238	2E-78
masp36	642	213	KT222310	MASP	TcCLB.508871.60	338	9E-118	MASP	TCDM_10876	355	2E-123	MASP	TCSYLVIO_009410	324	1E-111	MASP	Tc_MARK_5257	206	9E-66
masp37	648	215	KT222311	MASP	TcCLB.506757.50	328	1E-113	MASP	TCDM_08758	321	1E-110	MASP	TCSYLVIO_008640	325	1E-112	MASP	Tc_MARK_5257	237	5E-78
masp38	219	73	KT222312	Hypothetical protein*	TcCLB.509205.50	26.9	1.40	Hypothetical protein	TCDM_09521	57.8	2E-11	Hypothetical protein*	TCSYLVIO_008612	38.9	1E-04	Unspecified product	Tc_MARK_8690	50.1	2E-08

* Orthologs with e-value higher than 10^{-08} (the cutoff established for BLAST searches)

Table S4- List of MASP pseudogenes from the 3-step MASP library and their respective orthologs in the different strains

PSEUDOGENES	pb	aa (to 1st stop)	Genbank ID	CL Brener (esmeraldo, no-esm, CL)				Dm28c				Sylvio X10/1				Marinkellei strain B7			
				Annotation	Best Hit	Score	E-value	Annotation	Best Hit	Score	E-value	Annotation	Best Hit	Score	E-value	Annotation	Best Hit	Score	E-value
masp1.Ps	645	155	KT222313	MASP	TcCLB.510359.600	940	0.00	MASP	TCDM_08758	996	0.00	MASP	TCSYLVIO_008640	1009	0.00	MASP	Tc_MARK_5257	581	3E-165
masp2.Ps	773	4	KT222314	MASP pseudogene	TcCLB.506459.299	1272	0.00	MASP	TCDM_09835	168	8E-41	MASP	TCSYLVIO_008115	168	8E-41	MASP	Tc_MARK_6758	163	3E-39
masp3.Ps	1164	188	KT222315	MASP pseudogene	TcCLB.511643.73	1790	0.00	MASP	TCDM_11700	253	4E-66	MASP	TCSYLVIO_010405	798	0.0	MASP	Tc_MARK_10334	565	5E-160
masp4.Ps	778	59	KT222316	MASP pseudogene	TcCLB.506967.140	1011	0.00	MASP	TCDM_12282	672	0.0	MASP	TCSYLVIO_006664	356	3E-97	MASP	Tc_MARK_7257	600	1E-170
masp5.Ps	846	237	KT222317	MASP	TcCLB.508871.60	933	0.00	MASP	TCDM_10220	1050	0.00	MASP	TCSYLVIO_009410	1050	0.00	MASP	Tc_MARK_5257	497	1E-139
masp6.Ps	854	18	KT222318	MASP pseudogene	TcCLB.511605.5	601	5E-171	MASP	TCDM_12197	136	5E-31	MASP	TCSYLVIO_010758	122	1E-26	MASP	Tc_MARK_2180	71.6	2E-11
masp7.Ps	231	23	KT222319	MASP	TcCLB.508221.894	311	3E-84	MASP	TCDM_10614	264	4E-70	MASP	TCSYLVIO_010808	284	4E-76	MASP	Tc_MARK_3903	260	5E-69
masp8.Ps	454	26	KT222320	MASP	TcCLB.511255.280	634	0.00	MASP	TCDM_09622	726	0.00	MASP	TCSYLVIO_009298	246	2E-64	MASP	Tc_MARK_7492	219	3E-56
masp9.Ps	1063	39	KT222321	MASP pseudogene	TcCLB.511081.41	1420	0.00	MASP	TCDM_11444	168	1E-40	MASP	TCSYLVIO_009181	178	2E-43	MASP	Tc_MARK_6867	196	8E-49
masp10.Ps	776	130	KT222322	MASP pseudogene	TcCLB.506459.299	1274	0.00	MASP	TCDM_13341	134	2E-30	MASP	TCSYLVIO_007735	140	4E-32	MASP	Tc_MARK_6964	140	4E-32
masp11.Ps	1092	45	KT222323	MASP	TcCLB.507957.150	1112	0.0	MASP	TCDM_10560	1458	0.00	MASP	TCSYLVIO_006721	1276	0.00	MASP	Tc_MARK_5482	571	1E-161
masp12.Ps	645	155	KT222324	MASP	TcCLB.506765.50	928	0.00	MASP	TCDM_08758	933	0.00	MASP	TCSYLVIO_005169	957	0.00	MASP	Tc_MARK_5257	563	9E-160

* Orthologs with e-value higher than 10^{-08} (the cutoff established for BLAST searches)

Table S5- Distances obtained with MEGA6.06 among the members of the 3-step MASP library and the ortholog sequences identified in CL Brener. Dm28c. Sylvio X10/1 and Marinkellei strains of *T. cruzi*

Clone	CL Brener	DMc28	Sylvio X10/1	Marinkellei
masp1	0.177	0.126	0.098	0.477
masp2	0.177	0.126	0.093	0.477
masp3	0.157	0.100	0.083	0.511
masp4	0.131	0.103	0.103	0.458
masp5	0.178	0.131	0.203	0.411
masp6	0.148	0.098	0.103	0.482
masp7	0.157	0.100	0.083	0.511
masp8	0.156	0.065	0.461	0.582
masp9	0.254	0.097	0.351	0.646
masp10	0.304	0.177	0.482	0.534
masp11	0.190	0.168	0.438	0.542
masp12	0.201	0.182	0.182	0.182
masp13	0.141	0.779	0.585	0.677
masp14	0.150	0.156	0.210	0.421
masp15	0.157	0.145	0.157	0.431
masp16	0.186	0.174	0.242	0.462
masp17	0.407	0.085	0.105	0.726
masp18	0.129	0.123	0.175	0.403
masp19	0.129	0.129	0.181	0.403
masp20	0.403	0.389	0.250	0.732
masp21	0.219	0.128	0.405	0.527
masp22	0.157	0.100	0.083	0.511
masp23	0.152	0.141	0.135	0.436
masp24	0.177	0.065	0.405	0.566
masp25	0.152	0.152	0.212	0.433
masp26	0.129	0.123	0.175	0.403
masp27	0.216	0.096	0.074	0.434
masp28	0.413	1.197	1.128	0.413
masp29	0.181	0.110	0.468	0.667
masp30	0.135	0.129	0.135	0.413
masp31	0.248	0.142	0.125	0.466
masp32	0.139	0.128	0.168	0.408
masp33	0.258	0.239	0.202	0.488
masp34	0.176	0.141	0.188	0.390
masp35	0.140	0.129	0.175	0.403
masp36	0.166	0.082	0.104	0.526
masp37	0.129	0.123	0.175	0.403
masp1.Ps	0.327	0.070	0.048	0.269
masp2.Ps	1.449	1.172	1.137	1.333
masp4.Ps	0.110	0.218	0.401	0.261
masp5.Ps	1.101	1.149	1.113	1.213
masp6.Ps	0.126	1.668	1.285	1.740
masp7.Ps	0.150	0.222	0.217	0.242
masp8.Ps	1.070	1.073	0.909	0.789
masp9.Ps	0.143	0.651	0.693	0.608
masp10.Ps	0.044	8.855	6.117	7.049
masp11.Ps	0.136	0.066	0.089	0.327
masp12.Ps	0.089	0.095	0.077	0.243
